# The Interferon-Inducible Mouse Apolipoprotein L9 and Prohibitins Cooperate to Restrict Theiler’s Virus Replication

**DOI:** 10.1371/journal.pone.0133190

**Published:** 2015-07-21

**Authors:** Marguerite Kreit, Didier Vertommen, Laurent Gillet, Thomas Michiels

**Affiliations:** 1 Université catholique de Louvain, de Duve Institute, Brussels, Belgium; 2 Université de Liège, FARAH Research Center and Faculté de Médecine Vétérinaire, Liège, Belgium; University of Utah, UNITED STATES

## Abstract

*Apolipoprotein L9b* (*Apol9b*) is an interferon-stimulated gene (ISG) that has antiviral activity and is weakly expressed in primary mouse neurons as compared to other cell types. Here, we show that both *Apol9* isoforms (*Apol9b* and *Apol9a*) inhibit replication of Theiler’s murine encephalomyelitis virus (TMEV) but not replication of vesicular stomatitis virus (VSV), Murid herpesvirus-4 (MuHV-4), or infection by a lentiviral vector. *Apol9* genes are strongly expressed in mouse liver and, to a lesser extent, in pancreas, adipose tissue and intestine. Their expression is increased by type I interferon and viral infection. In contrast to genuine apolipoproteins that are involved in lipid transport, ApoL9 has an intracytoplasmic localization and does not seem to be secreted. The cytoplasmic localization of ApoL9 is in line with the observation that ApoL9 inhibits the replication step of TMEV infection. In contrast to human ApoL6, ApoL9 did not sensitize cells to apoptosis, in spite of the presence of a conserved putative BH3 domain, required for antiviral activity. ApoL9a and b isoforms interact with cellular prohibitin 1 (Phb1) and prohibitin 2 (Phb2) and this interaction might contribute to ApoL9 antiviral activity. Knocking down *Phb2* slightly increased TMEV replication, irrespective of ApoL9 overexpression. The antiviral activity of prohibitins against TMEV contrasts with the pro-viral activity of prohibitins observed for VSV and reported previously for Dengue 2 (DENV-2), Chikungunya (CHIKV) and influenza H5N1 viruses. ApoL9 is thus an example of ISG displaying a narrow antiviral range, which likely acts in complex with prohibitins to restrict TMEV replication.

## Introduction

Type I interferons (IFNs) mediate their antiviral effects through the expression of IFN-stimulated genes (ISGs). Recent studies based on large-scale gene knock down and overexpression screenings have evaluated the antiviral activity of hundreds of ISGs acting against RNA and DNA viruses [1–4]. Some ISG products display direct antiviral activity and sometimes act on a narrow virus range. Others act by regulating signal transduction pathways controlling IFN production and IFN responses and thus act on a broad range of viruses. The emerging picture is that a given virus is controlled by a specific range of ISGs, some of these ISGs being virus- or virus family-specific and others acting in a more general fashion.

We recently identified a group of mouse ISG that are not or weakly expressed in primary neurons after IFN-α/β treatment. Among these genes was the gene encoding apolipoprotein 9b (*Apol9b*). Apolipoproteins are typically associated with the transport of lipids in the organism. Accordingly, human apolipoprotein L1 (ApoL1) was originally described as a member of the high density lipoprotein family, which is involved in cholesterol transport [5]. However, the other members of the ApoL family were classified on the basis of sequence homology to ApoL1 but their functions may have diverged and remain to be characterized. The ApoL family is highly conserved across species [6]. *APOL* genes have been implicated in diseases such as schizophrenia and osteoarthritis, and are upregulated by both type I and type II IFNs [6, 7]. In human, six ApoL-coding genes (*APOL1*, *APOL2*, *APOL3*, *APOL4*, *APOL5* and *APOL6*) are clustered on chromosome 22q12 [8]. ApoL1 has been extensively studied and acts as a restriction factor for trypanosome infection [9]. In addition, ApoL1 and other human ApoL members (ApoL1, L2, L3 and L6) have emerged in high-throughput screenings of ISG activity, as proteins with antiviral activity against various classes of RNA viruses [1, 4]. ApoL1 restricted infection of cells by the AR86 strain of Sindbis virus and more modestly by Venezuelan equine encephalitis virus and human parainfluenza virus type 3, but increased infection by Yellow fever virus. ApoL2 slightly inhibited hepatitis C virus replication but had pro-viral activity toward Influenza A virus and respiratory syncytial virus (RSV). ApoL6 was reported to have antiviral activity against two picornaviruses, coxsackie B virus and poliovirus. ApoL6 also displays inhibitory activity against RSV [1, 4]. Overexpression of ApoL6 triggers apoptosis, which suggests that the antiviral effect of ApoL proteins may correlate with cell sensitization to apoptosis [10].

**Fig 1:**
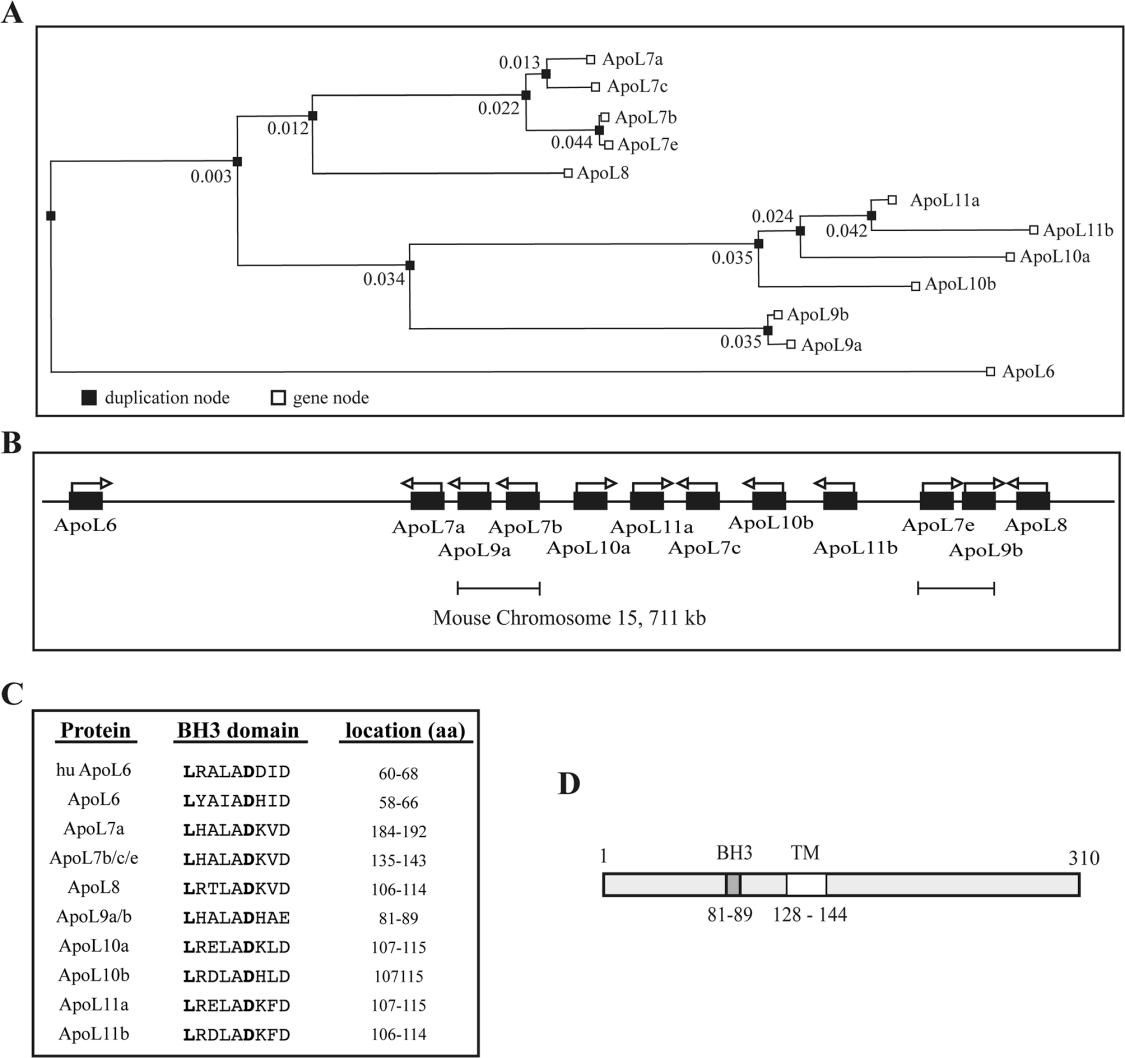
The murine ApoL family. A. Phylogenetic tree of the murine *Apol* gene family, generated by the Gene Orthology/Paralogy prediction method available on the Ensemble server (http://www.ensembl.org). Duplication nodes are indicated by black squares. Numbers represent the duplication confidence score. B. Organization of the mouse *Apol* genes cluster. Underlined regions (*Apol7b/9a* and *Apol7e/9b*) share 98% of nucleotide sequence identity and likely arose from a duplication event. C. Alignment of the human ApoL6 BH3 domain with the hypothetical BH3 domain of murine ApoL proteins. Two amino acids, *L* and *D*, shown in bold letters, are functionally conserved in all the BH3 domains identified. D. Structural organization of ApoL9 proteins. The putative BH3 domain (residues 81 to 89) is shown in dark gray. The white area (residues 128 to 144) corresponds to a potential transmembrane (TM) domain.

Mouse ApoL9b is a 310 amino acid-long protein, identical by 97% to ApoL9a. ApoL9a and ApoL9b (referred to collectively as ApoL9) are encoded by distinct genes. The murine Apolipoprotein L family is encoded by 12 genes and 1 pseudogene (*Apol6*, *Apol7a*, *Apol7b*, *Apol7c*, *Apol7e*, *Apol8*, *Apol9a*, *Apol9b*, *Apol10a*, *Apol10b*, *Apol11a*, *Apol11b* and pseudogene *Apol10c*) clustered on chromosome 15 (Fig 1). Murine ApoL6 is orthologous to human ApoL6, murine ApoL7a to human ApoL5, murine ApoL7b to human ApoL4 and murine ApoL8 to human ApoL2. It is unclear which human protein is orthologous to mouse ApoL9. We previously reported that ApoL9b is an antiviral ISG active against Theiler’s murine encephalomyelitis virus (TMEV or Theiler’s virus) [11]. The weak expression of *Apol9* in IFN-treated mouse primary neurons contributes to the surprising susceptibility of these cells to virus infection. This study aimed at defining the properties of murine ApoL9 proteins and at characterizing their antiviral functions.

## Materials and Methods

### Animal experiments

Ethics statement: Handling of mice (agreement LA1230472) and experimental procedures were conducted in accordance with the EEC directive 86/609/CEE and the related Belgian law of April 6th 2010. The study and protocol used in this study were approved by the ethics committee of the University of Louvain under the agreement # 2010/UCL/MD/031. To minimize the use of laboratory animals, *Apol9a* and *Apol9b* were amplified by RT-qPCR from samples of mouse RNA prepared for other experiments. RNA from organs of naïve C57BL/6 mice were obtained from Hermant et al [12]. Samples from FVB/N mice electro-injected with IFN-expressing and control plasmids were from Sommereyns et al. [13]. Brain and spinal cord RNA samples from TMEV-infected mice were obtained from 3 week-old FVB/N mice that were infected intracranially with 10^6^ PFU of the DA1 strain of TMEV for the indicated time (unpublished experiment). These mice were euthanized by deep anaesthesia (intraperitoneal administration of a 200 μl mix of Medetomidin hydrochlorid 300 mg/ml (Domitor) and Ketamine 1.5 g/ml (Anesketin)), and perfused with phosphate buffered saline, before tissue collection. Tissues were snap frozen in liquid nitrogen and kept at -80°C until RNA extraction.

### ApoL9 sequence analysis and bioinformatics

Murine ApoL cDNA and protein sequences were retrieved from the NCBI database [14]. Phylogenetic tree computation was generated by Ensembl [15]. TMbase was used to predict transmembrane domains [16], SignalP 4.1 to predict signal peptide [17] and SecretomeP to predict non-canonical secretory pathway [18]. CELLO [19] and SOSUI [20] were used to predict subcellular localization. Gene expression data and IFN-responsiveness of other members of the ApoL family were obtained from the interferome server [21].

### Cell culture, transfections and Brefeldin A treatment

L929 cells (ATCC), Neuro-2A (ECACC), HeLa-M [22, 23] and 293T cells [24] were maintained in Dulbecco's modified Eagle medium (Lonza) supplemented with 10% fetal calf serum (FCS) (Sigma) and 100U/ml of penicillin/streptomycin (Lonza). BHK-21 cells (ATCC) were cultured in Glasgow's modified Eagle's medium (GMEM) (Sigma) supplemented with 10% newborn bovine serum (Gibco), 100 U/ml of penicillin/streptomycin (Lonza), and 2.6 g of tryptose phosphate broth per liter (Difco).

Plasmid transfections were performed using *Trans*IT-LT1 transfection reagent (Mirus), according to the manufacturer’s instructions. For lentivirus production and co-immunoprecipitation experiments, 293T cells were transfected by the calcium phosphate method.

Transfection of the TM973 replicon RNA was performed as follows. The pTM973 plasmid carrying the replicon cDNA was linearized with restriction enzyme *ClaI* and subjected to *in vitro* transcription with T7 RNA polymerase, using the ribomax T7 kit (Promega, P1300). 0.5 μg of replicon RNA was then transfected in L929 cells (30,000 cells per well seeded in 24-well plates) with 1 μl of mRNA transfection reagent and 1 μl of booster reagent (Mirus, MIR 2250).

For Brefeldin A treatment, GolgiPlug (ref 555029, BD Biosciences) was diluted 1000-fold in the culture medium 20 hours after transfection of the cells and 4 hours prior to cell collection for western blot analysis.

### Interferon

IFN-β was produced and quantified, as described previously, from 293T cells transfected with pcDNA3-IFN-β [25], and diluted in culture medium at 5 units of IFN per ml for cell treatment.

### Viruses, cell infections and plaque assay

Viruses are presented in Table 1. Theiler's murine encephalomyelitis viruses (TMEV—or Theiler's virus) used in this study were KJ7 and KJ26, GFP/eGFP-expressing derivatives of the persistent DA1 strain and of the neurovirulent GDVII strain, respectively [11]. These viruses contain capsids adapted to infect L929 cells efficiently [26]. KJ7 and KJ26 were produced, as described [27, 28], from the corresponding full-length cDNA clones pKJ7 and pKJ26. The TMEV replicon expressing the firefly luciferase (called TM973) was constructed by replacing the L-capsid-part of 2A region (nucleotides 1064 to 3925 of pTMDA1-accession JX443418 [29]), by a DNA segment assembled by PCR and carrying in frame: i) the firefly luciferase ORF PCR-amplified from pGL4.16 (Promega); ii) the 2A coding sequence of FMDV which enables co-translational dissociation of the protein segment translated downstream of this sequence [30], and iii) the CRE replication signal of pTMDA1 [31]. The VSV (Strain Indiana) derivative expressing eGFP from an additional transcription unit located between the G and L genes was a gift from Martin Schwemmle (Univ. of Freiburg, Germany) [32]. The MuHV-4 derivative used in this study contains the eGFP gene in a bacterial artificial chromosome cassette [33] and was generated and purified as described previously [34]. Viruses used in this study were produced and titrated on BHK-21 cells.

**Table 1:** Recombinant viruses used in the study.

**Plasmid/Virus** ^1^	**Parental virus/vector**	**Characteristics** ^2^
(pTM)DA1	TMEV (DA1)	molecular clone of wild type DA strain of TMEV
(p)KJ7	TMEV (DA1)	GFP-coding sequence replacing codons 5–67 of L; capsid adapted to infect L929 cells
(p)KJ26	TMEV (GDVII)	eGFP-coding sequence replacing codons 1–67 of L
(p)TM973	TMEV (DA1)	TMEV replicon expressing the firefly luciferase
MuHV-4-GFP	MuHV-4	eGFP-coding sequence in a BAC cassette
VSV-GFP	VSV	eGFP-coding sequence between G and L genes

1. virus DA1 is produced by reverse genetics from plasmid pTMDA1. Viruses KJ6 and KJ7 are produced from pKJ6 and pKJ7 respectively

2. L = leader protein of Theiler's virus; eGFP = enhanced GFP

For flow cytometry analysis, infection of L929 cells was performed in the following conditions: KJ7: 0.25 PFU per cell for 8.5 hours; KJ26: 0.5 PFU per cell for 10 hours; VSV-GFP: 4 PFU per cell for 7 hours; MuHV-4: 4 PFU per cell for 18 hours. For infectious virus yield analysis, infection conditions were: KJ7: 0.25 PFU per cell for 20 hours; KJ26: 0.5 PFU per cell for 20 hours; VSV-GFP: 4 PFU per cell for 20 hours; MuHV-4: 4 PFU per cell for 28 hours. After infection, cells underwent two freeze (-80°C)/thaw cycles. Then, virus released in the cell supernatant was quantified by standard plaque assay on BHK-21 cells. Plaques formed by GFP-expressing viruses were counted under the fluorescent microscope, one or two days post-infection for VSV, three days post-infection for TMEV, and five days post-infection for MuHV-4.

### Lentiviral vectors and cell transduction

Lentiviral vectors used in this study are listed in Table 2. Lentiviral vectors used for RNA interference are described in the RNA interference section. Lentiviral vectors used for protein expression were derived from pCCLsin.PPT.hPGK.GFP.pre. [35]. pTM944 is a derivative of this vector, which carries the eGFP coding sequence translated from the TMEV IRES. Transcription of the IRES-eGFP region is driven by the cytomegalovirus (CMV) promoter.

**Table 2:** Recombinant lentiviral vectors used in the study.

**Plasmid/Virus** ^1^	**Parental**	**Characteristics** ^2^
(p)TM942	pCCLsin	Lentiviral vector carrying: PGK->MCS-IRES-mCherry
(p)TM943	pCCLsin	Lentiviral vector carrying: CMV->MCS
(p)TM944	pCCLsin	Lentiviral vector carrying: CMV->MCS-IRES-eGFP
(p)TM945	pCCLsin	Lentiviral vector carrying: CMV->MCS-IRES-mCherry
(p)MK44	pTM942	PGK-> ApoL9b-IRES-mCherry
(p)MK65	pTM943	CMV-> FLAG-ApoL9b3
(p)MK66	pTM943	CMV-> ApoL9b-FLAG4
(p)MK82	pTM945	CMV-> FLAG-ApoL9b-IRES-mCherry
(p)MK83	pTM945	CMV-> ApoL9b-FLAG-IRES-mCherry
(p)MK85	pTM942	PGK-> ApoL9a-IRES-mCherry
(p)MK86	pTM943	CMV-> FLAG-L*5
(p)MK87	pTM943	CMV-> FLAG-ApoL9a3
(p)MK88	pTM943	CMV->ApoL9a- FLAG4
(p)MK89	pTM942	PGK-> ApoL9b D50 N-terminal residues-IRES-mCherry
(p)MK90	pTM942	PGK-> ApoL9a D50 N-terminal residues-IRES-mCherry
(p)MK95	pTM942	PGK-> ApoL9b D50 C-terminal residues-IRES-mCherry
(p)MK96	pTM942	PGK-> ApoL9a D50 C-terminal residues-IRES-mCherry
(p)MK101	pTM942	PGK-> ApoL9b with mutated BH3-IRES-mCherry
(p)MK102	pTM942	PGK-> ApoL9a with mutated BH3-IRES-mCherry
(p)MK117	pLKO.1	shRNA empty vector with E2-Crimson-coding region replacing Puro^R^
(p)MK118	pMK117	shRNA targeting Phb1 coding region and co-expression E2-Crimson
(p)MK119	pMK117	shRNA targeting Phb1 coding region and co-expression E2-Crimson
(p)MK120	pMK117	shRNA targeting Phb2 coding region and co-expression E2-Crimson
(p)MK121	pMK117	shRNA targeting Phb2 3'UTR and co-expression E2-Crimson

1. the names of plasmids used to produce the listed lentiviral vectors contain an extra p (e.g. pMK44 for lentivirus MK44)

2. pCCLsin = pCCLsin.PPT.hPGK.GFP.pre

3. Sequence of the FLAG-Apol9 junction is: MG-DYKDDDDK-GS-MASS… where GS is a linker and MASS are the N-terminal 4 residues of ApoL9.

4. Sequence of the Apol9-FLAG junction is: …YKTI-GS-DYKDDDDK where GS is a linker and YKTI are the C-terminal 4 residues of ApoL9.

5. L* = L star protein of Theiler’s virus; PGK = human phosphoglycerate kinase promoter; CMV = cytomegalovirus promoter; MCS = multi-cloning site; eGFP = enhanced GFP; Puro^R^ = puromycin resistance; Δ = deletion

*Apol9* constructs were cloned in lentiviral vectors pTM942, pTM943 or pTM945. pTM942 is bicistronic vector carrying a phosphoglycerate kinase (PGK) promoter, a multi-cloning site, the IRES of TMEV and the mCherry coding sequence [36]. pTM943 contains the CMV promoter and a multi-cloning site. pTM945 is similar to pTM942 but contains the CMV promoter instead of the PGK promoter.

To construct these vectors, the coding sequences of *Apol9b* and *Apol9a* were PCR-amplified from C57BL/6 mouse spleen cDNA, using Phusion polymerase (Finnzymes) and cloned in pTM942 using *Age*I/*Bsi*WI restrictions sites introduced in the PCR primers.

Vectors encoding FLAG-tagged constructs and deletion mutants of *Apol9* were obtained by PCR amplification of this gene with primers carrying the FLAG-coding sequence and *Age*I and *Bsi*WI restrictions sites that were used to clone the PCR fragment in the lentiviral vectors (Table 2).

The region coding the putative BH3 domain of ApoL9 was mutated by a two-step PCR with overlapping divergent primers used to introduce the mutations. The amino acid sequence of BH3 was mutated from LHALADHAE to GHAGAAHGE for ApoL9a and GHAGAAHAE for ApoL9b.

In all the constructs, the fragments obtained by PCR and their boundaries with the vectors were sequenced to rule out unexpected mutations. Lentivirus particles were produced, as described previously [11], by transient transfection of 293T cells. Transduction of cells were performed by adding filtered, lentivirus stock in the culture medium of cells seeded at low confluence.

The plasmid used to express C-terminally FLAG-tagged mouse IFN-αA is pcDNA3-IFN-αA-FLAG (or pAGE2) [12].

### RNA interference

pMK117 is a pLKO.1 [37] derivative constructed by substituting the E2-Crimson coding region for that of the puromycin resistance gene. E2-Crimson was amplified by PCR from pEF1alpha-E2-Crimson vector (Clontech, 63181) with primers introducing *Bam*HI and *Kpn*I restrictions site and cloned into pLKO.1. shRNA sequences targeting the mouse *Phb1* and *Phb2* transcripts were selected from the RNA mission collection (Sigma) and cloned in pMK117 using *Age*I and *Eco*RI restriction sites introduced in the PCR primers. Constructs pMK118 (shRNA TRCN0000312198) and pMK120 (shRNA TRCN0000302352), targeting coding sequences of *Phb1* and *Phb2* respectively, gave a more complete inhibition than constructs pMK119 and pMK121 that express shRNA TRCN0000088454 and TRCN0000054428, targeting the coding sequence of *Phb1* and the 3’UTR of *Phb2*, respectively (not shown). pMK118 and pMK120 were thus used for subsequent knock down experiments. Stocks of lentiviral vectors derived from pLKO.1 were generated, as described previously [11]. Efficiency of RNA silencing was assayed 3 days after transduction, by evaluating Phb1 and Phb2 protein expression by Western blot.

### Measurement of apoptosis and luciferase assay

Caspase 3/7 activity was measured using the Caspase-Glo 3/7 Assay (Promega, G811A). Briefly, 10,000 L929 cells grown in 96 well plates were left untreated, infected with 1 PFU per cell of KJ26 for 10 hours or treated with 1uM staurosporine (Sigma, S-4400) for 7 hours to induce cell apoptosis [38]. After plate equilibration at room temperature, 50 μl of Caspase-Glo 3/7 reagent was added to each well. The plate was placed on a plate shaker, in the dark, for one hour at room temperature and luminescence was measured with a plate-reading GloMax luminometer (Promega). Raw data are presented as relative light units (RLU).

In samples from L929 cells transfected with the TM973 replicon, firefly luciferase activity was measured using the luciferase assay system (Promega, E1501), according to the manufacturer’s instructions. Luminescence was measured with a GloMax 20/20 luminometer (Promega).

### Co-immunoprecipitation assays and western blotting

Protein extracts were prepared from 293T cells, 24 hours post-transfection, as previously described [39]. FLAG-tagged ApoL9 and IFN-α were detected by western blot using sodium dodecyl sulfate-polyacrylamide gel electrophoresis (SDS-PAGE) gels containing 12% acrylamide and run in a Tris-Tricine buffer. Blots were probed with anti-FLAG polyclonal (Covance, PRB-132P) and anti-β-actin monoclonal (Sigma, A5441) antibodies.

Co-immunoprecipitation assays were conducted on transfected 293T or Neuro-2A cells, or on transduced L929 cells, as previously described [29]. Proteins were immunoprecipitated with anti-FLAG (Covance, PRB-132P), anti-Phb1 (SantaCruz, sc-28259) or anti-Phb2 (SantaCruz, sc-67045) polyclonal antibodies and proteins were detected using SDS-PAGE and immunoblot analysis.

### Protein identification by mass spectrometry

After co-immunoprecipitation, samples were resolved using a 10% Tris-Glycine SDS-PAGE and proteins were visualized using PageBlue (Thermo Scientific, 24620). Bands of interest were cut out from the gel and digested with trypsin (50ng/μl in 50 mM NH4HCO3 buffer, PH 8.0). The peptides were analyzed, as previously described [40], by capillary LC-tandem mass spectrometry in a LTQ XL ion trap mass spectrometer (ThermoScientific, San Jose, CA) fitted with a microelectrospray probe. The data were analyzed with the ProteomeDiscoverer software (ThermoScientific, version 1.4.1), and the proteins were identified with SequestHT against a target-decoy nonredundant human or mouse proteins database obtained from Uniprot. The following parameters were used: trypsin was selected with proteolytic cleavage only after arginine and lysine, number of internal cleavage sites was set to 1, mass tolerance for precursors and fragment ions was 1.0 Da, considered dynamic modifications were + 15.99 Da for oxidized methionine. Peptide matches were filtered using the q-value and Posterior Error Probability calculated by the Percolator algorithm ensuring an estimated false positive rate below 5%. The filtered Sequest HT output files for each peptide were grouped according to the protein from which they were derived.

### RNA extraction and quantitative RT-qPCR

Total RNA preparations, reverse transcription (RT) and quantitative PCR (qPCR) reactions were performed as previously described [41]. qPCR standards consisted of 10-fold dilutions of quantified plasmids carrying the sequence to be amplified: pTM793 for *β-actin*, pMK72 for *Apol9b*, pMK68 for *Apol9a* and pCS40 for *Oasl2*. These plasmids are derivatives of pCR4-ToPo (Invitrogen) in which the corresponding PCR fragments were cloned. Real-time PCR primer sequences and reference plasmid used are given in Table 3.

**Table 3:** primers used for gene expression analysis by RT-qPCR.

**Gene/virus** ^1^	**Primer**	**Sequence 5’→ 3’**	**Ref. plasmid** ^2^
TMEV (s)	TM348	GAA CGT CAG CAT TTT CCG GC	pTM410
TMEV (as)	TM349	GGT GTG AAG AGC GGC AAG TG	pTM410
β (s)	TM421	AGA GGG AAA TCG TGC GTG AC	pTM793
β (as)	TM422	CAA TAG TGA TGA CCT GGC CGT	pTM793
ApoL9b (s)	TM1479	GTA GCT AGG ATT GTC AAC AAG A	pMK72
ApoL9b (as)	TM1513	CAG AGG GGT TCC TTC CAG CC	pMK72
Oasl2 (s)	TM638	GGA TGC CTG GGA GAG AAT CG	pCS40
Oasl2 (as)	TM639	TCG CCT GCT CTT CGA AAC TG	pCS40
ApoL9a (s)	TM1464	GTG GAT AGG ATT GCC AGC AAG	pMK68
ApoL9a (as)	TM1465	AGA GGG GTT CCT TTC AGA CTG	pMK68

1. (s)sense and (as) antisense

2. Ref. plasmids: reference plasmids containing the corresponding amplicons and used for quantitative PCR.

### Immunolabeling and microscopy

HeLa-M cells were fixed for 15 min with PBS containing 4% of paraformaldehyde (PFA). Cells were then permeabilized for 5 min with PBS containing 0.1% of Triton X-100. Blocking occurred for 1 h at room temperature in TNB blocking reagent (Perkin Elmer). Cells were next incubated with the anti-FLAG mouse monoclonal antibody (Sigma-Aldrich, F3165; 1/500) diluted in TNB for one hour. AlexaFluor-488 conjugated secondary antibody (Invitrogen) was incubated for 1 h at a 1/800 dilution in TNB. Finally, cells were mounted with Mowiol for fluorescence microscopy. Antibodies used for double labeling were: anti-transferrin receptor (Abcam mouse monoclonal, ref Ab5339); anti-dsRNA (K1 and J2 mouse monoclonal, English and scientific consulting Bt); anti-TMEV VP1 protein (mouse monoclonal F12B3, [11]); anti-PBodies (mouse monoclonal anti-p70 S6 kinase, Santa Cruz ref sc-8418 [42]). For nuclear staining, cells were incubated for 5 min in Hoechst 33258 before the final washing steps. Lipid dropplets were stained at the same step, by incubating the cells for 15 in BODIPY 493503 diluted 1/1000 (Life Technologies). The Golgi compartment was identified after staining glycosylated proteins with Alexafluor564-conjugated wheat germ agglutinin (Molecular probes–W11262). The endoplasmic reticulum was identified by co-transfection of pDsRed-ER as performed in [12]. For staining the endosomal pathway, cells were incubated for 60 min before fixation with 1 mg/ml of 10kDa Dextran-Alexafluor594 (Life technologies D-22913). For mitochondrial staining, cells were incubated for 45 min before fixation with 200 nM mitotracker red FM (Life technologies M-22425). Fluorescence microscopy was performed using a spinning disk confocal microscope equipped with an Axiocam MRm camera (Zeiss). Intensity, contrast, and color balance of images were equilibrated using Zen (Zeiss) and Adobe Photoshop.

### Flow cytometry

After dissociation with trypsin-EDTA, cells were suspended in PBS buffer containing 5% of FCS and 0.5% of paraformaldehyde. Data acquisition was done with a LSR Fortessa flow cytometer (BD Bioscience) using the FACSDiva software. Data were analyzed using FlowJow 9.6.4. as follows: i) single cells were gated with side and forward scatters; ii) cells transduced with ApoL9 or shRNA-expressing vectors were gated as mCherry or E2-Crimson-positive cells; iii) in these gated cell populations, infection efficiency of GFP-expressing viruses was calculated as the percentage of GFP-positive cells multiplied by the mean GFP fluorescence intensity (MFI) of these cells. Normalization was done by dividing the infection efficiency of each population by the average infection efficiency of cell populations transduced with the empty vector (= ratio to negative control).

### Statistical analyses

Results are expressed as mean +/- standard deviations (SD). Statistical significance was analyzed using the two-tailed Mann-Withney *U* test. NS is indicative of p-values higher than 0.05; * p-value < 0.05; ** p-value < 0.01; *** p-value < 0.001

## Results

### Mouse ApoL9 genes and proteins

Mouse *Apol* genes appear to derive from a common ancestor (Fig 1A) and are clustered on chromosome 15 (Fig 1B). *Apol9a and Apol9b* genes are likely issued from a gene duplication event, since the region encompassing the *Apol7b* and *Apol9a* genes shares 98% nucleotides identity with that encompassing the *Apol7e* and *Apol9b* genes (Fig 1B). Murine ApoL proteins possess a conserved domain named ApoL (referred as pfam05461) whose function is still unknown. Previously, human ApoL6 was reported to exert a pro-apoptotic activity through a Bcl-2 homology (BH) domain, designated BH3 [10]. Sequence alignments predict the presence of BH3 domains in several mouse ApoL proteins, including ApoL9 (Fig 1C) [43]. ApoL9 is mostly hydrophilic but contains a short hydrophobic region (residues 128 to 144), compatible with a transmembrane domain (Fig 1D).

### ApoL9 are cytoplasmic proteins

**Fig 2:**
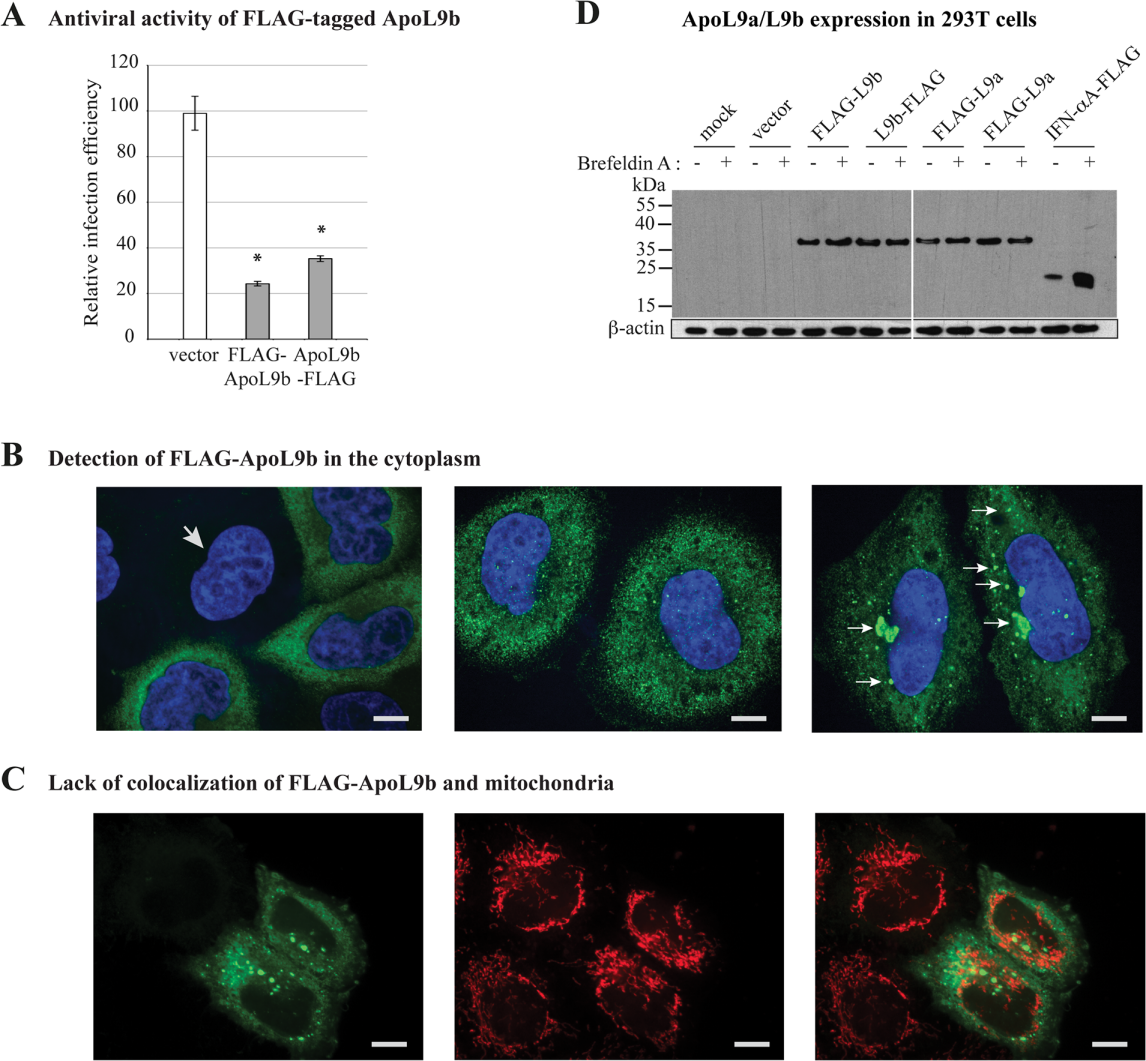
ApoL9 proteins are not-secreted, cytoplasmic proteins. A. Antiviral activity of N-terminally (FLAG-ApoL9b) and C-terminally (ApoL9b-FLAG) FLAG-tagged ApoL9b against TMEV. Histograms show the relative infection efficiency, of L929 cells that were transduced with theTM945 lentiviral vector expressing mCherry alone (vector), or with the MK82 or MK83 derivatives co-expressing mCherry and FLAG-ApoL9b or ApoL9b-FLAG, respectively. Infection efficiency was measured, by flow cytometry, after infection of cells for 10 hours with 0.5 PFU per cell of the GFP-expressing KJ26 virus. B. FLAG detection (green) and nuclear staining (blue) in HeLa-M cells transduced with the MK65 construct expressing FLAG-ApoL9b (scale bar: 10 um). Typical immunolabeling patterns are shown: left and central panels: one non-transduced cell (arrow) and transduced cells showing a diffuse cytoplasmic FLAG-ApoL9b localization; right panel: cells showing typical small and medium-sized FLAG-ApoL9b granules (arrows). C. Confocal images showing FLAG (green—left panel) and mitochondria (red—central panel) labelings and merged image (right panel). Nuclei were stained with Hoechst (Blue). D. Western blot detection of FLAG-tagged ApoL9b, ApoL9a and IFN-αA in 293T cells. Cells were mock-transfected, transfected with the empty pTM943 plasmid (vector), or with pTM943 derivatives coding for the indicated FLAG-tagged ApoL9 proteins. As a control cells were also transfected with plasmid pcDNA3-IFNαA-FLAG, which expresses the secreted FLAG-tagged mouse IFN-αA [12]. 20 hours post-transfection, cells were mock-treated (-) or treated for 4 hours with 10ug/ml of Brefeldin A (+) prior to total protein extraction. FLAG and β-actin were detected by Western blot in cell lysates.

Since anti-ApoL9 antibodies were not available, we constructed vectors encoding N- and C-terminally FLAG-tagged ApoL9b, to define the subcellular localization of the protein by FLAG immunodetection. First, we confirmed that N-terminally and C-terminally FLAG-tagged ApoL9b conserved the ability to exert an antiviral activity against TMEV in L929 cells (Fig 2A). Next, FLAG-tagged ApoL9b was detected by immunofluorescence in HeLa-M cells transduced with lentiviral constructs. ApoL9 localization was similar in cells transduced with pMK65 and pMK66, expressing N- and C-terminally tagged ApoL9b proteins respectively (not shown). We observed a diffuse cytoplasmic labeling and accumulation of ApoL9b as granule-like structures in part of the cells (Fig 2B). ApoL9b granules did not co-localize with markers for mitochondria (Fig 2C), endoplasmic reticulum, endosomes, lysosomes, P-bodies, Golgi vesicles, and lipid droplets or with viral components such as double-stranded RNA or VP1 capsid antigen (data not shown). Interestingly, in contrast to prototypic apolipoproteins, which are secreted, ApoL9 does not contain any predicted signal sequence (Signal P scores were 0.119 for ApoL9a and 0.133 for ApoL9b –cutoff value was 0.5) nor is it predicted to be secreted by a non-canonical secretory pathway by the Secretome P server. Absence of ApoL9 secretion by the classical secretion pathway was further confirmed by showing that Brefeldin A treatment did not trigger accumulation of N- or C-terminally FLAG-tagged ApoL9 proteins in transfected 293T cells, in contrast to C-terminally FLAG-tagged IFN-α that was taken as a secreted protein control (Fig 2D). We conclude that ApoL9 are cytoplasmic proteins that are not secreted, at least not by the classical secretion pathway.

### Tissue distribution of *Apol9a* and *Apol9b* expression and induction by type I IFN

**Fig 3:**
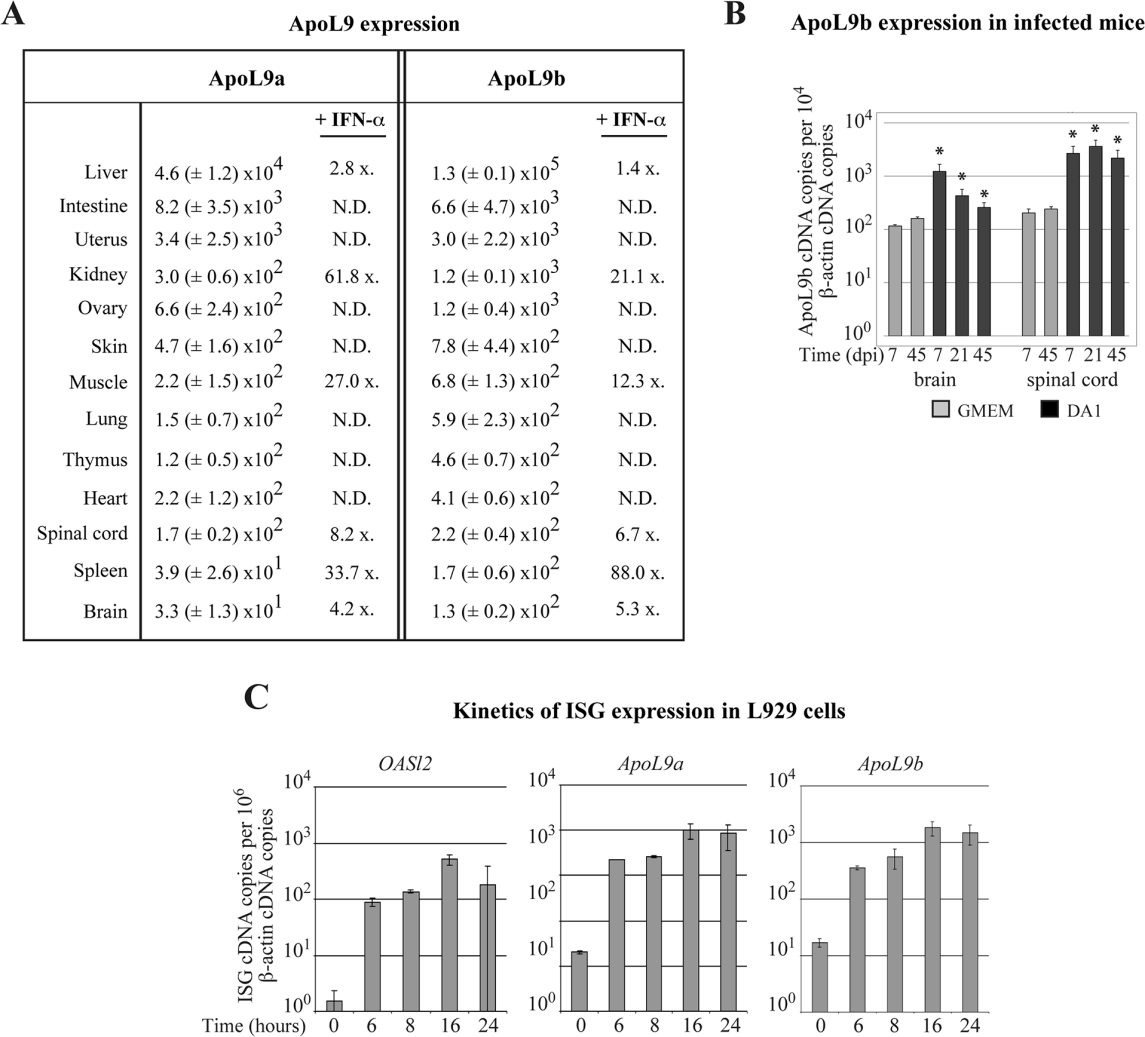
*Apol9* expression in mouse tissues and in L929 cells. *Apol9* transcripts were quantified by RT-qPCR in mouse tissues and cells. A. *Apol9a* and *Apol9b* cDNA copies (mean and SD) detected per 10^6^ β-actin cDNA copies, in tissues of naive C57BL/6 mice (n = 3). The column indicated “+ IFN-α” gives the mean upregulation (x-fold) of *Apol9* expression observed in the presence of systemic IFN-α. This corresponds to the ratio between the number of *Apol9* cDNA copies detected in tissues of FVB/N mice electro-injected in the tibialis muscle with plasmid pcDNA3-IFN-α6T, which expresses IFN-α that circulates in the blood stream (n = 6) and that of control mice that were electro-injected with an empty pcDNA3 plasmid. N.D. = not determined. B. *Apol9b* expression in the central nervous system of FVB/N mice that were mock-infected (n = 3) or infected intracerebrally with 10^6^ PFU of the DA1 strain of TMEV (n = 5) for the indicated time. *: indicates a significant difference with the mock-infected mice at 7dpi. C. Time course of *Oasl2*, *Apol9a* and *Apol9b expression* in L929 cells treated with 5 units/ml of IFN-β (n = 3).

Expression patterns of *Apol9a* and *Apol9b* in mouse organs were very similar, in agreement with the fact that their putative gene promoter sequences were highly conserved. In untreated mice, both genes were predominantly expressed in the liver, and to a lesser extent, in pancreas, adipose tissue and intestine (Fig 3A). Circulating IFN-α triggered *Apol9* upregulation in many tissues (Fig 3A). In the liver, upregulation of *Apol9* expression by IFN-α was less clear, likely because basal expression of *Apol9* was already very high in this organ. In the central nervous system (CNS), the induction of *Apol9* was also moderate, presumably because *ApoL9* gene transcription is low in neurons and because circulating IFN-α did not cross the blood brain barrier. However, *Apol9* expression was upregulated in the brain and spinal cord after infection with Theiler’s virus, likely because of local type I IFN production (Fig 3B). Accordingly, screening of the GEO microarray database [44] confirmed that *Apol9* expression increased with viral infection, *in vivo*, in many mouse tissues (Table 4). In L929 fibroblasts, time course of *Apol9a* and *Apol9b* gene induction after IFN-β stimulation was comparable to that of *Oasl2* (Fig 3C). Upregulation of *Apol9* gene transcription was almost maximal from 6 hours post-treatment, suggesting that *Apol9* expression is directly induced by type I IFN. Taken together, our data indicate that *Apol9a and Apol9b* are *bona fide* ISGs that share a similar expression profile. Intriguingly, in spite of the unusual cytoplasmic nature of these apolipoproteins, they are mainly produced by liver cells, like typical apolipoproteins.

**Table 4:** Upregulation of ApoL9a/b expression in microarray data sets.

**Virus**	**Tissue / Cell type**	**Time point**	**Fold change**	**ref**
Coxsackie virus B3	Heart	D10	4.6 (females) / 3.7 (males)	[55]
Rabies virus (CVS-11)	Brain / Spinal cord	D7	6.3 / 6.5	[56]
Japanese encephalitis virus	Neuro-2A cells	D2	9.0	GEO: GSE20135
Vaccinia virus	Lungs	D3 / D4 / D5	2.9 / 17.4 / 21.5	[57]
Sendai virus	BMDC cells	D1	14.4	[58]
H1N1 Influenza A/Mexico/4108/2009	Lungs	D1 / D2 / D3 / D4 / D5 / D6	3.0 / 11.7 / 9.6 / 9.3 / 10.9 / 12.2	[59]
H1N1 Influenza A/California/04/2009	Lungs	D3 / D5	5.3 / 9.0	[60]
H5N1 Influenza A/Vietnam/1203/04	Lungs	D2	26	GEO: GSE40792
SARS Coronavirus	Lungs	D4 / D7	13.6 / 4.0	GEO: GSE40827
Mink enteritis virus	Brain	D10	3.5	GEO: GSE42264

### ApoL9 has a limited antiviral range

**Fig 4:**
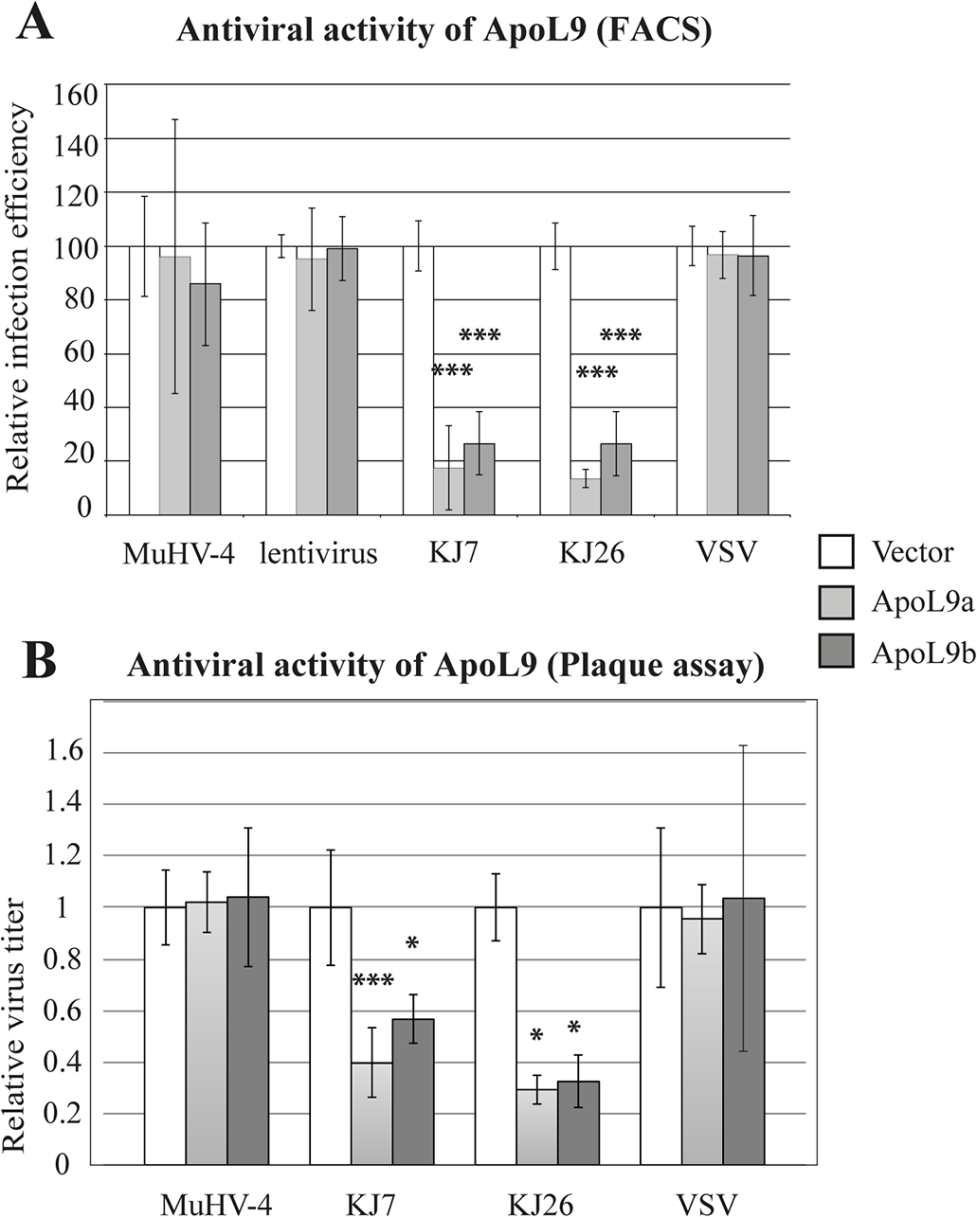
ApoL9 has antiviral activity against a restricted virus range. L929 cells were transduced with the TM942 vector expressing mCherry alone (Vector/ white columns), or with the MK85 (light grey columns) and MK44 (dark grey columns) derivatives of this vector, that express Apol9a and Apol9b respectively. Three days after transduction, cells were infected with the indicated GFP-expressing viruses. Titer of the TM944 GFP-expression lentiviral vector was adjusted to transduce about 30% of cells. A. Relative infection efficiency (mean and SD), as determined by flow cytometry, for indicated GFP-expressing viruses. Infection conditions (MOI and time) were: KJ7: 0.25–8.5 hpi; KJ26: 0.5–10hpi; VSV-GFP: 4–7hpi; MuHV-4: 4–18hpi. Titer of the TM944 GFP-expression lentiviral vector was adjusted to transduce about 30% of cells and analyzed 2 days after transduction. Values were pooled from three independent experiments performed each with triplicate infection samples (n = 9). B. Infectious virus titers (mean and SD) measured by plaque assay for indicated GFP-expressing viruses. Infection conditions (MOI and time) were: KJ7: 0.25–20 hpi; KJ26: 0.5–20hpi; VSV-GFP: 4–20hpi; MuHV-4: 4–28hpi. Virus titers were normalized to those generated in cells transduced with the empty vector (n = 4 for cells transduced with MK44 or infected with KJ26. n = 8 for other conditions). *: significance of the difference between cells expressing ApoL9 and cells carrying the empty vector.

Antiviral activity of ApoL9 was examined, by flow cytometry, using GFP-expressing viruses representative of different virus families: i) KJ7 and KJ26, which are derived from persistent and neurovirulent Theiler’s virus strains respectively (*Picornaviridae*, positive-stranded RNA viruses); ii) vesicular stomatitis virus (VSV) (*Rhabdoviridae*, negative-stranded RNA virus); iii) murid herpesvirus-4 (MuHV-4)(*Herpesviridae*, DNA virus); and iv) TM944, a lentiviral vector derived from HIV (*Retroviridae*, *RNA/DNA virus*). L929 cells were first transduced with lentiviral vectors pMK85 and pMK44 co-expressing *Apol9a* or *Apol9b* and mCherry, or with the control vector, pTM942, expressing mCherry alone. Three days after transduction, cells were infected with the various GFP-expressing viruses and analyzed by flow cytometry. Replication of the various viruses (GFP fluorescence) was analyzed in the population of transduced (mCherry-positive) cells. As shown in Fig 4A, *Apol9a* and *Apol9b* expression significantly inhibited infection by both persistent and neurovirulent TMEV strains but had little impact, if any, on the other tested viruses. To confirm these results, infectious virus yield was examined by plaque assay. Therefore, cells were transduced with the same *Apol9*-expressing lentiviral vectors. Three days after transduction, mCherry-positive cells were sorted and cultured for 2 days before infection with the GFP-expressing TMEV, MuHV-4 and VSV derivatives. Infectious virus yield was measured by plaque assay (Fig 4B). Again, ApoL9 expression significantly reduced TMEV but not MuHV-4 or VSV production. These data confirm the restricted virus range of ApoL9 antiviral activity.

### ApoL9 does not act by sensitizing cells to apoptosis

**Fig 5:**
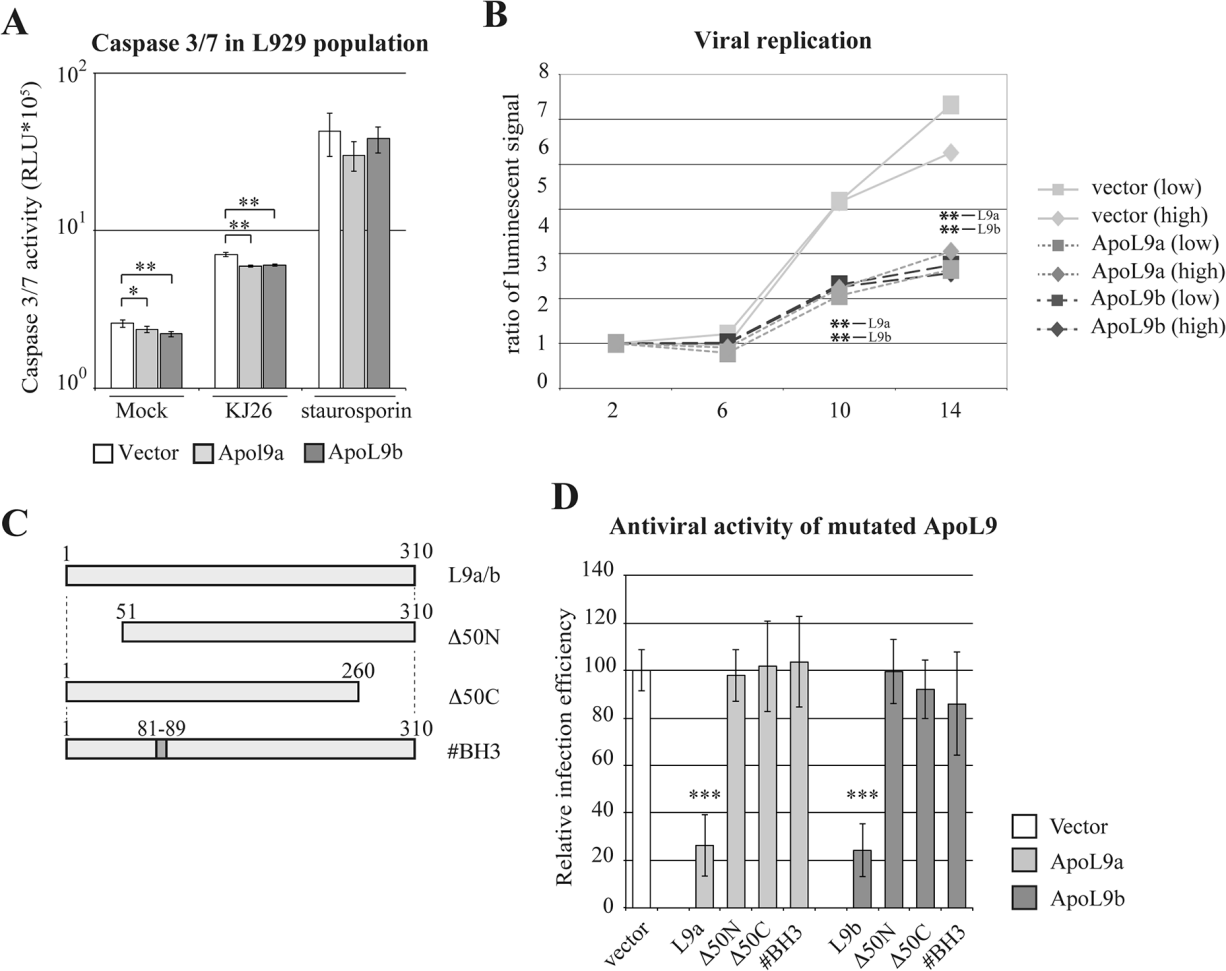
ApoL9 does not sensitize cells to apoptosis but acts on TMEV replication. A. Histograms show caspase 3 and 7 activities (mean and SD), measured in L929 cell populations that were transduced with the empty TM942 vector (white columns) or with the MK85 (light grey columns) and MK44 (dark grey columns) derivatives that express Apol9a and Apol9b respectively. These cells were mock-treated (Mock), infected with 1 PFU per cell of TMEV (KJ26) for 10 hours or treated with 1uM staurosporine for 7 hours (n = 3). B. Luciferase activity produced by a TMEV-luciferase replicon (TM973) transfected in ApoL9 expressing cells. Prior to replicon transfection, L929 cells were transduced with TM942 (vector-mCherry), MK85 (co-expression of mCherry and ApoL9a) or mK44 (co-expression of mCherry and ApoL9a). mCherry-positive cells were FACS sorted and subdivided in population of high (> 10^4^) and low (4.10^3^ to 10^4^) mCherry fluorescence. *In vitro* transcribed RNA of the TM973 replicon was transfected in these cells and luciferase activity was assayed at the indicated times post-transfection. Graphs show the luciferase activity normalized, for each construct, to the activity measured at two hours post-transfection. Results are representative of a typical experiment out of 3 performed in triplicate. * indicate the significance of the difference between cells expressing ApoL9 and the empty vector. C. Schematic representation of ApoL9 mutants. #BH3 refers to point mutations introduced in the putative BH3 domain. Δ50N and Δ50C refer to the deletion of the 50 N- and C-terminal amino acids, respectively. D. relative infection efficiency of L929 cells transduced either with the empty TM942 vector (vector) or with the same vector encoding wild type or mutant ApoL9 proteins (ApoL9a in light gray and ApoL9b in dark gray). Cells were infected for 8.5 hours with 0.5 PFU per cell of virus KJ7. GFP fluorescence was examined in cells that were gated for expression of the ApoL9 constructs (mCherry-positive). Histograms show the mean and SD, for a pool of 3 independent infection experiments performed in triplicates. For each experiment, the infection efficiency in ApoL9-expressing cells was normalized to that measured in cells carrying the empty vector. Stars indicate the difference significance between ApoL9-expressing cells and cells carrying the empty vector.

Human ApoL6 was reported to induce apoptosis when overexpressed in cells, most likely through its BH3 domain [10, 45]. Since this domain is well conserved in other ApoL proteins including ApoL9 (Fig 1C), we tested whether ApoL9a and ApoL9b proteins could exert their antiviral activity by sensitizing cells to apoptosis. Apoptosis was measured with a caspase 3/7 assay, in L929 cells that were transduced with an empty vector or with lentiviral vectors expressing *Apol9a* or *Apol9b* (Fig 5A). In the absence of TMEV infection, ApoL9 expression did not increase but rather triggered a minor decrease of caspase activity. Staurosporine strongly increased caspase activity as expected, irrespective of ApoL9 expression. Infection of cells with TMEV induced apoptosis. However, cells expressing *Apol9* were slightly but significantly less sensitive to virus-induced apoptosis than control cells (Fig 5A). This result excludes that ApoL9 may act by sensitizing infected cells to apoptosis. In contrast, ApoL9 appears to restrict viral infection and thereby to limit virus-induced apoptosis. Similar results were obtained by comparing caspase activity in three *Apol9b*-overexpressing and three control L929 cell clones obtained previously [11] (data not shown). In conclusion, contrary to the reported pro-apoptotic activity of human ApoL6, the antiviral effect of ApoL9 does appear to be mediated by a pro-apoptotic influence of ApoL9.

### ApoL9 acts on TMEV replication

To test whether ApoL9 acts on entry or on replication of TMEV, we used a TMEV replicon (TM973) expressing the firefly luciferase. Replicon RNA was transfected into cells to bypass the virus entry step and luciferase activity was followed as a surrogate marker of TMEV replication. Replicon-derived luciferase activity was measured in L929 cells transduced with the empty pTM942 vector or with the pTM942 derivatives expressing ApoL9 (Fig 5B). From 10 hours post-transfection, luciferase activity was significantly lower in all ApoL9-expressing cell populations. We conclude that ApoL9 restricts the replication of TMEV.

### ApoL9 regions required for antiviral activity

We then generated L929 cells that overexpress ApoL9 mutants in order to map the ApoL9 domains required for antiviral activity (Fig 5C). For both *Apol9a* and *Apol9b*, deletion of either the C- or the N-terminal 50 residues of ApoL9 abrogated antiviral activity as did the mutation of the putative BH3 domain (amino acids 81–89) (Fig 5D). These results suggest that mutations may have affected the ApoL9 conformation required for antiviral activity.

### ApoL9 interacts with cellular prohibitin-1 and -2

**Fig 6:**
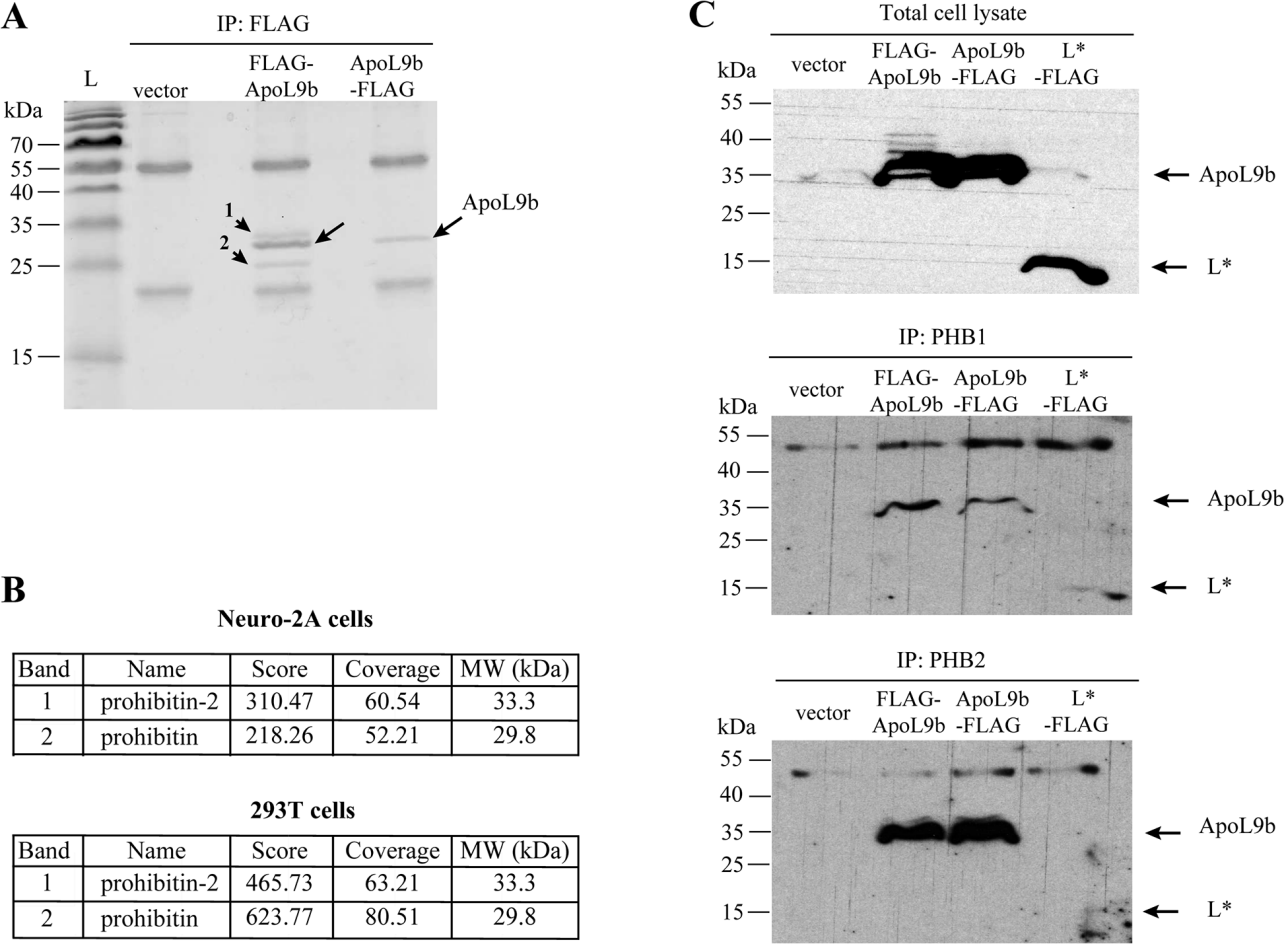
ApoL9 interacts with prohibitins. A. Coomassie blue-stained gel of ApoL9b co-immunoprecipitation products. Samples were co-immunoprecipitated with an anti-FLAG antibody from 293T cells that were transfected with the pTM943 empty vector or with MK65 and MK66 expressing N- or C-terminally FLAG-tagged ApoL9b, respectively. Long arrows point to the tagged ApoL9 bands and short arrows, numbered 1 and 2 indicate co-immunoprecipitated products. Note that bands 1 and 2 were also visible but at a lower intensity in the ApoL9b-FLAG sample (right lane). L is the protein molecular weight ladder. B. Proteins corresponding to bands 1 and 2, as identified by mass spectrometry in mouse (Neuro-2A) and human (293T) cells. C. Co-immunoprecipitation of ApoL9 with Phb1 and Phb2. Western blot detection of FLAG-tagged ApoL9b after immunoprecipitation of endogenous Phb1 or Phb2 proteins. Lysates were prepared from 293T cells transfected with the empty pTM943 vector, with pTM943 derivatives expressing ApoL9b-FLAG or FLAG-ApoL9b and, as a negative control, with plasmid pMK86 expressing a N-terminally FLAG-tagged L* protein of TMEV. The upper panel shows FLAG detection in cell lysates corresponding to 5% of the input used for immunoprecipitations. Central and lower panels show FLAG detection after immunoprecipitation of Phb1 and Phb2, respectively.

Next, we investigated whether ApoL9 would act by interacting with cellular proteins. Therefore, mouse (Neuro-2A) and human (293T) cells were transfected with vectors allowing the expression of N- or C-terminally FLAG-tagged ApoL9b. Cellular partners of ApoL9b were identified 24 hours post-transfection, by co-immunoprecipitation and mass spectrometry. Two protein bands were detectable on Comassie blue-stained gels loaded with products that co-precipitated with ApoL9b in both Neuro-2A and 293T cells (Fig 6A and data not shown). For both mouse and human cells, bands 1 and 2 were identified as prohibitin 1 and -2 (Phb1 and Phb2) respectively (Fig 6B and S1 Dataset). Prohibitins (PHBs) 1 and 2 are two ubiquitous multifunctional proteins that may act as chaperones (reviewed by Thuaud, Ribeiro [46]). Interaction between ApoL9b and PHBs was further confirmed by detection of FL AG-ApoL9b and ApoL9b-FLAG after immunoprecipitation of endogenous Phb1 or Phb2 proteins from cells expressing the FLAG-tagged ApoL9 proteins (Fig 6C). In these experiments, a N-terminally FLAG-tagged TMEV L* protein, used as negative control, was not (Phb1) or very weakly (Phb2) co-immunoprecipitated with prohibitins, supporting the specificity of the ApoL9-prohibitin interaction. The experiment could, however, not be confirmed with endogenous ApoL9 proteins, given the lack of anti-ApoL9 antibodies.

### ApoL9 and PHBs contribute to antiviral activity

**Fig 7:**
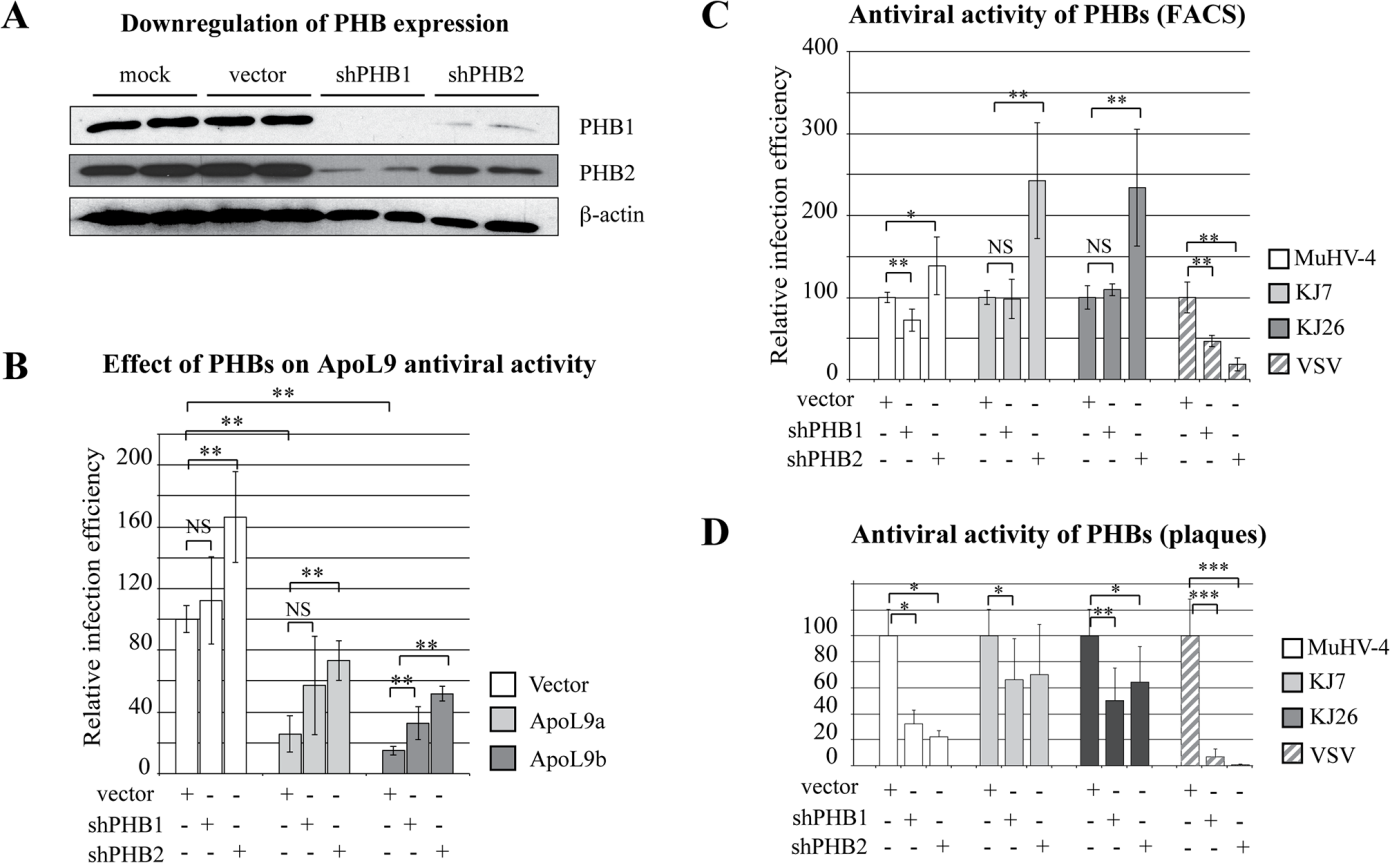
Prohibitins modulate viral infection. A. Western blot showing the detection of endogenous Phb1 and Phb2 proteins, as well as β-actin in L929 cells (Mock) transduced with the empty vector MK117 (vector) or with MK117 derivatives expressing shRNA directed against PHB1 (MK118) or against PHB2 (MK120). B. Influence of prohibitins on ApoL9-mediated inhibition of TMEV replication. L929 cells were transduced with the TM942 vector expressing mCherry alone (white) or with TM942 derivatives, MK85 and MK44, co-expressing mCherry and ApoL9a (light gray) or ApoL9b (dark gray). These cell populations were subsequently transduced with vectors expressing E2-Crimson alone (vector) or co-expressing E2-Crimson and shRNAs targeting *Phb1* (MK118) or *Phb2* (MK120). Three days after transduction with the shRNA vectors, cells were infected for 8.5 hours with 0.25 PFU per cell of the GFP-expressing virus KJ7 and infection was analyzed by flow cytometry. Viral replication (GFP) was examined in cells that were gated for expression of the indicated constructs (ApoL9-mCherry or shRNA-E2-Crimson). Histograms show the relative infection efficiencies (mean and SD) for 2 independent infection experiments performed each in triplicate (n = 6). In each experiment, the infection efficiency in ApoL9-expressing cells was normalized to that measured in cells carrying TM942 and the empty shRNA vector. Stars denote significant differences between PHB knocked down and control cells. C. Influence of prohibitins on replication of TMEV, MuHV-4 and VSV. L929 cells transduced with MK117 derivatives expressing E2-Crimson alone (vector) or co-expressing E2-Crimson and a shRNAs targeting *Phb1* (MK118) or *Phb2* (MK120) were infected with GFP-expressing viruses: MuHV-4, TMEV (KJ7 or KJ26), and VSV. Infection conditions (MOI and time) were: KJ7: 0.25–8.5 hpi; KJ26: 0.5–10hpi; VSV-GFP: 4 – 7hpi; MuHV-4: 4 – 18hpi. Histograms show the relative infection efficiencies (mean and SD), measured by flow cytometry, of a pool of 2 experiments performed with triplicate infections. For each experiment, the infection efficiency in shRNA-expressing cells was normalized to that measured in cells carrying the empty vector. D. Influence of prohibitins on infectious virus yield. Infectious virus yield was examined by plaque assay in infected cells that were transduced as in C. Infection conditions (MOI and time) were: KJ7: 0.25–20 hpi; KJ26: 0.5–20hpi; VSV-GFP: 4 – 20hpi; MuHV-4: 4 – 28hpi. Histograms show the mean and SD of the virus titers produced by PHB knocked down cells normalized to that produced by cells carrying the empty vector (n = 4 for MuHV-4; n = 8 for other viruses). Stars denote significant differences as compared to the empty vector and NS non-significant differences.

To determine whether association with PHBs is necessary for ApoL9 antiviral activity, we used a *Phb* knock down strategy. L929 cells transduced to co-express mCherry and *Apol9b* or Apo*l9a*, or mCherry alone (vector), were subsequently transduced with lentiviral vectors co-expressing shRNAs targeting the coding sequence of either *Phb1* or *Phb2* and the far-red fluorescent E2-Crimson protein [47], prior to infection with the GFP-expressing KJ7 virus. Knock down of PHBs expression was confirmed by western blot (Fig 7A and data not shown). As reported previously [48], knocking down either *Phb1* or *Phb2* triggered a strong reduction in the amount of both Phb proteins, presumably because stability of Phb1 and Phb2 depends on their mutual interaction. It is worth noting that PHBs knock down triggered a cell growth inhibition from about 5 days post-transduction, which prevented the use of sorted pure populations of PHB knock down cells. Thus, three days after the transduction of ApoL9-expressing cells with vectors expressing shRNA directed against PHBs or control vectors, cells were infected with the GFP-expressing KJ7 virus and analyzed by flow cytometry.

As in previous experiments, overexpression of both *Apol9a* and *Apol9b* decreased KJ7 replication. Knocking down *Phb2* and, to a lesser extent, *Phb1* expression in cells that overexpressed *Apol9* significantly enhanced but did not fully restore infection (Fig 7B). Interestingly, knocking down *Phb*2 also increased infection by TMEV in cells that did not overexpress *Apol9*, suggesting that this prohibitin has antiviral activity *per se*, against TMEV. This increase of TMEV infection after knock down of *Phb* gene expression was unexpected. Indeed, PHBs were previously reported to act as pro-viral factors that facilitate Dengue 2 (DENV-2), Chikungunya (CHIKV) and H5N1 virus entry in cells [48–50]. We therefore compared the influence of *Phb* knock down on infection by TMEV (KJ7 or KJ26), MuHV-4, and VSV, using flow cytometry (Fig 7C). While *Phb2* knock down reproducibly favored the infection by both strains of TMEV, it failed to significantly affect MuHV-4 infection and even inhibited VSV infection. We also monitored virus yield, by plaque assay, after PHBs knock down. For this purpose, cells were transduced with the shRNA-expressing lentiviral vectors, at concentrations calculated to yield 90–95% of transduction. Three days after transduction, cell populations were infected with VSV, MuHV-4 and TMEV and virus yield was measured by plaque assay (Fig 7D). This experiment confirmed the strong pro-viral activity of PHBs toward VSV but failed to show the anti-viral effect of PHBs toward TMEV. The discrepancy between the highly reproducible negative effect exerted by PHBs against TMEV infection, measured by flow cytometry and the positive effect on infectious virus particles production may be the consequence of contrasting roles of PHBs at different steps of the virus infection cycle (pro-viral for entry and anti-viral for replication). Alternatively, it may be an indirect effect of the progressive cell growth inhibition that occurs over time, after PHBs knock down.

## Discussion

Our data show that *Apol9a* and *Apol9b* are two antiviral ISGs that are constitutively expressed in mouse tissues such as liver, pancreas, adipose tissue and intestine. Expression of these genes can be further upregulated by type I IFN. Both ApoL9 isoforms display antiviral activity against Theiler’s virus but not against VSV, MuHV-4 or a lentiviral vector derived from HIV-1. ApoL9 thus belongs to the group of ISG displaying a narrow antiviral range.

Other members of the *Apol* gene family (*Apol6*, *l7a*, *l7c*, *l9a* and *l9b)* are upregulated by type I and type II IFN but have different tissue distributions. *Apol7a* is highly expressed in the liver, like *Apol9*, while *Apol6* is mainly expressed in adipose tissue and mammary glands and *Apol7c* in CD8+ cells and lymph nodes [21]. The function of these proteins is still elusive. From their IFN-inducible property, it can be anticipated that they also act as antiviral proteins and, given their tissue distribution, that they contribute to shape the tissue tropism of viruses.

In contrast to ApoL1, ApoL9 lacks a typical signal sequence. We failed to detect FLAG-tagged ApoL9 in the supernatant of cells transfected with constructs expressing either N-terminally or C-terminally tagged proteins (data not shown) and brefeldin A treatment did not lead to ApoL9 intracellular accumulation (Fig 2D). Furthermore, immunofluorescent detection did not show any association of ApoL9 with components of the secretory pathway, such as the endoplasmic reticulum or the Golgi apparatus. Our interpretation of these data is that ApoL9 is not secreted, at least not by the classical secretory pathway. This conclusion contrasts with the recent report of Sun et al. who reported that ApoL9 may be secreted by macrophages to enhance epithelial cell proliferation [51]. To reconcile these observations, one could hypothesize that ApoL9 proteins may be secreted in a cell type-specific fashion.

In line with a cytoplasmic localization of ApoL9, this protein was found to inhibit the replication of a TMEV replicon that was transfected into cells. ApoL9 did not, however, act by sensitizing cells to apoptosis. ApoL9 immunolocalization did not provide a clear hint toward the mode of action of this protein. In part of the cells, ApoL9 formed granule-like structures. In the absence of anti-ApoL9 antibodies to detect the endogenous protein, we cannot prove that these granules are formed in physiological conditions. However, a recent work reports the appearance of similar granules in the case of human ApoL2 [52]. Neither ApoL2 nor ApoL9 granules colocalized with tested subcellular compartments. Nevertheless, most ApoL9 molecules were detected as diffuse cytoplasmic proteins and it is therefore difficult to rule out their association with specific cell components or with components of virus replication complexes.

By co-immunoprecipitation, we found that ApoL9 interacts with cellular prohibitins Phb1 and Phb2, both in mouse and human cells. PHBs are highly conserved and widely expressed proteins, which are present in many different cellular compartments including the cytoplasm, mitochondria and cell surface [53, 54]. They interact with many proteins, including viral proteins and are sometimes considered as chaperones (reviewed by Thuaud, Ribeiro [46]). Knocking down *Phb2* expression did not completely abrogate the antiviral protection conferred by ApoL9 overexpression (Fig 7B). This suggests that PHBs association with apoL9 is not strictly required for ApoL9 antiviral activity. Alternatively, the incomplete abrogation of ApoL9 activity may result from incomplete *Phb* knock down. It is tempting to speculate that PHBs may act as chaperones to stimulate the antiviral activity of ApoL9. More surprisingly, *Phb2* knock down slightly but reproducibly increased cell susceptibility to TMEV replication, independently of ApoL9 expression. This suggests that PHBs may, *per se*, have an antiviral activity. This is contrasting with the strong proviral effect of PHBs observed for VSV (Fig 7B and 7C) Dengue virus 2 (DENV-2), Chikungunya virus (CHIKV) and H5N1 influenza virus [48–50].

In conclusion, our data suggest that ApoL9a and ApoL9b are two IFN-induced cytoplasmic proteins that antagonize TMEV by cooperating with prohibitins. These apolipoproteins largely differ from genuine lipid transport-associated lipoproteins, both by their intracytoplasmic localization and by their antiviral function. Why ApoL9 isoforms are particularly weakly expressed in neurons is still unclear.

## Supporting Information

S1 Dataset ApoL9-interacting proteins. Excel file showing the complete set of proteins identified by mass spectrometry, in bands 1 and 2 of Commassie blue-stained gels (see Fig 6A) loaded with FLAG-ApoL9 immunoprecipitates from Neuro-2A and 293T cells.
(XLSX)
